# The power of observation in clinical medicine

**DOI:** 10.5116/ijme.5fb9.1c9b

**Published:** 2020-11-30

**Authors:** Fabrizia Faustinella

**Affiliations:** 1 Department of Family and Community Medicine, Baylor College of Medicine, Houston, TX, Houston, USA

**Keywords:** Observation, clinical medicine, USA

## To the Editor

Clinical observation and inspection are fundamental to the practice of medicine. They are central aspects of the physical examination as they lead to accurate diagnoses and treatment. The terms observation and inspection are often used interchangeably, although inspection refers specifically to what we can observe visually on the surface of the body, while observation is a broader term that refers to the careful use of our senses to gain physical as well as behavioral information. Something that is immediately visible when evaluating a patient is the color of the skin. This can provide important clues to the underlying medical problem and guide the clinician towards a diagnosis. Suffice to mention the skin pallor in anemia, the yellowing of the skin in jaundice, the bluish hue seen in cyanosis, the cherry red skin color of carbon monoxide poisoning, the patchy loss of skin color in vitiligo, the mottled, marbled appearance of the skin in livido reticularis, the orange skin pigmentation of carotenemia, among many others.

While working at one of our walk-in clinics, I saw a patient who presented with complaints of fatigue, weight loss and abdominal pain. The man, whom I will name Juan, was very concerned because his symptoms were impairing his ability to work and earn a living. He had previous history of histoplasmosis and was unclear if he had received proper treatment for it or not. I continued interviewing the patient while perusing his electronic medical record (EMR), my eyes darting back and forth from him to the computer screen and vice versa.   I saw multiple visits in several outpatient primary clinics, walk-in clinics,  and emergency departments, all with similar complaints. A number of tests and imaging studies were done, but the various clues to his disease didn't appear to have been tied together at any point in time. It occurred to me that his skin had an unusual tan.   I started looking at him more intently,   my eyes shifting up and down from his face to his hands.   The knuckles were very dark compared to the surrounding skin. I approached him, and on further physical examination, I noticed hyper-pigmented gingival patches, black longitudinal nail bands and darkened palmar creases.   The patient's wife became aware of my intense scrutiny of Juan's hands and face, and rightfully guessed that I must have been puzzled by the color of his skin.

"Doctor, my husband's skin was much lighter, just like mine," she said in Spanish, her native language, while pointing at her own face. She added that his color had changed during the past several months, becoming progressively darker.

That was the clue, purely based on clinical observation, which allowed the diagnosis to be made even prior to further workup.

In Juan's case, the hyperpigmentation of the skin unmistakably pointed towards the diagnosis of adrenal insufficiency,  especially once considered in association with the other less specific symptoms of fatigue,  weight loss and abdominal pain.  In addition to that,  several tests found in the  EMR showed electrolytes abnormalities and   MRI  findings of bilateral enlargement of the adrenal glands, which are seen in disseminated TB, fungal infections, lymphoma and other conditions, all resulting, if untreated,  in adrenal insufficiency.  The MRI was one of the many imaging studies requested for further workup of the abdominal pain, but nobody seemed to have paid attention to the enlarged adrenals at any of the various office visits performed by different health care professionals.

I referred Juan to my endocrinology colleagues for an expedited evaluation.  Further tests were performed and he was officially diagnosed with primary adrenal insufficiency due to disseminated histoplasmosis.  Juan was treated accordingly and he's now thriving again.

Yet, how could Juan's diagnosis be missed for so long? Granted that the onset of adrenal insufficiency is often insidious and nonspecific, how is it possible that nobody paid attention to the striking skin discoloration and the mounting constellation of signs and symptoms? Also, despite the availability of various abnormal tests and lab reports in the EMR pointing towards the possibility of adrenal insufficiency, there was still a significant delay in diagnosis.  This delay in diagnosis can be explained by several factors which are the results of fundamental changes in the practice of medicine and medical education during the past decades:  lack of continuity of care, poor communication among health care providers compounded by the use of different EMR systems,  and a general decline in clinical skills.  The continuity of clinical information is in large part dependent on the continuity of the physician-patient relationship,^1^^-^^2^ but nowadays patients often end up seeing different providers:   rotating residents and medical students under the supervision of various attending physicians, nurse practitioners, physician assistants, community clinic physicians at various locations in town, walk-in clinics and emergency departments. The involvement of multiple providers can profoundly affect the continuity of care, even if the EMR system is usually the same, at least within university-affiliated health care facilities, allowing for easy access to all the available data. The fragmentation is, of course, even greater when different EMRs are used, as so very often is the case.

Primary care physicians are also more and more pressed for time, with increasing patient panels and shorter office visits, making it more challenging for them to attentively listen to their patients, to carefully review the record, to gather all pertinent information, and share that information with other colleagues involved in the care of those patients.^3^The other contributing factor is, without any doubt, a general decline in clinical skills such as history-taking skills, physical examination skills, and clinical reasoning skills which all require a solid fund of knowledge, practice, and experience.  Oversights in history-taking and physical examination, including observation, can lead to delayed diagnosis, unnecessary tests and treatment, escalating medical costs, and potentially life-threatening consequences for patients, as exemplified by Juan's medical mishap.

There is a consensus that clinical skills have been greatly deteriorating during the past twenty years, with some reports dating back to the 1970s and 1980s.^4^^-^^6^ The problem of deteriorating clinical skills has gained worldwide attention through an increase in publications and discussion panels.^7^^-^^8^ The reasons behind this deterioration are very complex and represent a challenge for our academic and medical institutions, as I have outlined in a previous publication.^9^

Juan's case also reveals how the decline in clinical skills and the progressive fragmentation of care can lead to a loss of the immense value associated with knowing, talking,  and listening to the patient, getting a good history, and performing an accurate physical exam. This close and considerate attention is how the physician and the patient establish a rapport of trust and respect, which transcends any other considerations. Such trust and mutual respect should be at the heart of the practice of medicine.

### Conflict of Interest

The authors declare that they have no conflict of interest.
